# Hypertension in Cameroon associated with high likelihood of obstructive sleep apnea: a pilot study

**DOI:** 10.1186/s12872-017-0542-1

**Published:** 2017-05-08

**Authors:** Alfred Kongnyu Njamnshi, Michel Karngong Mengnjo, Eta Ngole Mbong, Samuel Kingue, Julius Yundze Fonsah, Andreas Ateke Njoh, Leonard Njamnshi Nfor, Leonard Ngarka, Samuel Eric Chokote, Felicien Enyime Ntone

**Affiliations:** 1 Faculty of Medicine and Biomedical Sciences, University of Yaoundé 1, Yaounde, Cameroon; 2 c/o PC Great Soppo, P.O. Box 547, Buea, Cameroon

**Keywords:** Sleep, Sleep questionnaires, Obstructive sleep apnea, hypertension, Cameroon

## Abstract

**Background:**

Although disordered sleep patterns predispose to hypertension (HTN), little is known on the effect of the latter on sleep patterns in sub-Saharan Africa. This study therefore sought to generate preliminary data on the likelihood (risk) of Obstructive sleep apnea (OSA) in hypertensive patients, with the aid of sleep questionnaires.

**Methods:**

This case–control study, age-and-sex-matched HTN patients with normotensive participants, and compared sleep patterns in either group determined with the aid of the Berlin Questionnaire (BQ) and Epworth Sleepiness Scale (ESS).

**Results:**

Overall, 50 HTN and 54 age- and sex-matched normotensive participants were enrolled. The prevalence of snoring was higher in participants with hypertension compared to normotensives (58.0% versus 44.0% respectively), though not significantly, (*p* = 0.167). However, the hypertensive cases (aged on average 54.78 ± 8.79 years and with mean duration since diagnosis of 4.46 ± 4.36 years) had a significantly higher likelihood of Obstructive Sleep Apnea (OSA) than the controls (aOR = 5.03; 95% CI, 1.90–13.33, *p* = 0.001) and but no significant resulting daytime sleepiness (*p* = 0.421). There was no clear trend observed between both the risk of OSA and daytime sleepiness and HTN severity. Although not significant, participants with controlled hypertension had lower rates of risk of OSA compared to those with uncontrolled HTN (50.0% versus 63.2%, *p* = 0.718).

**Conclusions:**

Preliminary findings of this study (the first of its kind in Cameroon) suggests that hypertension is positively associated with likelihood of OSA in Cameroon. Further studies are required to investigate this further and the role of sleep questionnaires in our setting, cheap and easy to use tools which can be used to identify early, patients with hypertension in need for further sleep investigations. This will contribute to improving their quality of life and adherence to anti-hypertension treatment.

## Background

Hypertension (HTN) is a growing worldwide [1] and national public health challenge for which several health surveys in developed and developing countries have revealed a high prevalence, a large percentage of them poorly controlled [2–4]. The disease occurs as a result of an interaction between environmental, genetic, social and lifestyle factors [5, 6], requiring multifactorial and multidisciplinary management approach for favorable outcomes.

Sleep plays an important role in the regulation of body processes and as such contributes significantly to the quality of life in humans [7]. Though known to be a restorative and refreshing process, over the past 50 years habitual sleep duration has decreased by 1.5 to 2 h/day, due to the evolving lifestyle and standards of living [8]. Sleep is characterized amongst other events by complex activity of the cardiovascular autonomic mechanisms and consequent changes in arterial pressure and heart rate [8]. Poor sleep has been recognized as independent risk factor of hypertension [9, 10] as it if often characterized by recurrent episodes of intermittent hypoxia. There is growing evidence that obstructive sleep apnea (OSA) a form of Sleep Disordered Breathing (SDB) is common among patients with hypertension, ranging from 37% [11] to 56% [12] as well as evidence that managing sleep disordered breathing improves blood pressure (BP) values and response to hypertensive treatment [13].

In Cameroon, sleep studies are few [14], and to our knowledge, no study so far has been carried out to assess sleep in hypertensive participants. It was hence essential to conduct assessments of sleep patterns of hypertensive patients in Cameroon with the aid of clinician-friendly, readily-available, less costly and easy-to-use sleep questionnaires, in order to generate preliminary data on the likelihood (risk) of obstructive sleep patterns (and consequent daytime sleepiness) in patients with hypertension compared to normotensive participants from which further exploration, management and awareness-raising initiatives can be conducted.

## Methods

### Study design

A case–control study carried in 50 consenting hypertensive participants age- and sex-matched with 54 normotensive participants.

### Study setting and ethical considerations

The study was carried out in Yaoundé, the cosmopolitan capital city of Cameroon, specifically at the out-patient unit of the cardiology and neurology departments of the Yaoundé Central Hospital, a government run tertiary health facility which serves as referral centre for Yaoundé and its environs. In addition, the Neurology Department of this hospital has the lone sleep laboratory in the country that supports this kind of study.

Ethical approval was obtained from the institutional review board of the Faculty of Medicine and Biomedical Sciences (FMBS) of the University of Yaoundé 1, and administrative clearance from the directorate of the Yaoundé Central Hospital. Study participants provided informed consent and information they provided and patient files were coded and confidentially handled.

### Study participants

Cases (hypertensive persons) included in the study were participants aged ≥ 18 years followed-up at the outpatient unit of the YCH, with mean blood pressure (BP) ≥140/90 mmHg or an optimal BP control, and who were on at least one anti-hypertensive drug. Individuals with abnormal sleep-wake cycles due to night-shift work, medication, history of tracheotomy, current home oxygen therapy (Continuous Positive Airway Pressure) treatment, as well as hypertensives patients with evidence of secondary hypertension, patients with history of target organ damage such as stroke, renal failure or myocardial infarction (obtained either during clerking or information gotten from patient files) and pregnant women were excluded.

For each case, a consenting normotensive participant (mean systolic blood pressure, SBP ≤ 140 mmHg and mean diastolic blood pressure, DBP ≤ 90 mmHg), of same age and sex, was enrolled from the community as control. A total of 113 participants were enrolled but at the time of analyses, 9 participants (5 participants with co-morbidity and 4 with incomplete data) were excluded giving a total of 104 participants (50 hypertensive and 54 normotensive participants).

### Study instruments

Socio-demographic and clinical data of study participants were collected through interviews (in French and English, the languages commonly spoken by Cameroon’s numerous ethnic groups) with the aid of a pretested structured questionnaire. Data collected included age, sex, occupation, marital status, region of origin, address, history of diagnosis of hypertension, frequency of follow up, treatment strategy, number of current anti-hypertensive drugs, duration of treatment, cigarette smoking and alcohol status (for cases). All study participants underwent a complete physical examination with particular attention on neurological, cardiovascular and respiratory examination and anthropometric measurements.

The risk of obstructive sleep apnea was evaluated with the aid of the Berlin Questionnaire [15] and daytime sleepiness with the aid of the Epworth Sleepiness Scale [16].

Sleep quality (risk of OSA and Daytime sleepiness) was assessed and interpreted with the following scales as follows:

- *Scoring for OSA with the Berlin Questionnaire (BQ)*
  - If ≥ 2 positive categories: participant considered High Risk of OSA
  - If ≤ 1 positive category: participant considered Low Risk of OSA
- *Scoring for daytime sleepiness with Epworth Sleepiness Scale (ESS)*

The Epworth Sleepiness Scale is an 8-item questionnaire. Each question is graded from 0 to 3, depending on the likelihood to fall asleep during the day.

0- No chance of falling asleep,
1- Slight chance to fall asleep,
2- Moderate chances to fall asleep and
3- High chance to fall asleep.

The grading of the total ESS score as recommended by Rosenthal LD and Diana DC [17] was as follow:

- 1–7 = Good; getting restful sleep.
- 8–14 = Sleep debt.
- 15 or higher = Excessive daytime somnolence

### Definition of study variables

Exposure/independent variables:

- *Blood Pressure status*: Classified as hypertensive or normotensive based on the average BP measurements as well as past history of hypertension controlled or not with appropriate management.
- *Classification of hypertension*: Grouping into 4 stages according to the Joint National Committee (JNC) 8 staging algorithm [9] of blood pressure values obtained during clinical examination
  - Normal: Systolic BP < 120 mmHg and Diastolic BP < 80 mmHg
  - Pre-hypertension: Systolic BP = 120–139 mmHg or Diastolic BP = 80–89 mmHg
  - Stage 1 hypertension: Systolic BP = 140–159 mmHg or Diastolic BP = 90–99 mmHg
  - Stage 2 hypertension: Systolic BP ≥ 160 mmHg or Diastolic BP ≥ 100 mmHg
- *Treatment strategy*: Classified as either lifestyle modifications only, pharmacologic or both. *Year of diagnosis and disease duration*: Year of diagnosis reported by the patient (confirmed by diagnosis report) after which *disease duration* was determined after comparing with study date. The latter were expressed in years and months respectively then categorized ordinally as shown on Table 3.
- *Number of routine visits to physician per year*: As reported by study participants and confirmed by patient records. Assessed as continuous data and then ordinally categorized as shown on Table 2.
- *Number of anti-hypertensive drugs taken and compliance to treatment during the previous month* was assessed in numerical order. Hypertensive participants were considered compliant if they reported not having missed taking prescribed anti-hypertensive medications not more than 7 times in a month.
- *Signs and symptoms of anxiety and depression*: Determined using the 14 items Hospital anxiety and depression scale, which scores each item 0–3. A score of ≥11 is considered a case of depression.

Outcome/dependent variables:

- *Daytime sleepiness*: Ordinally categorized with respect to ESS score: score of 1–7 (restful sleep and no daytime sleepiness), score 8–14 (sleep debt), score ≥ 15 (excessive daytime somnolence).
- *Risk of OSA:* participants were considered as having a “high risk of OSA” if ≥2 categories were positive and “low risk of OSA” if ≤1category was positive.

### Study covariantes

Age: Reported by study participants and grouped into 3 age groups: 25–49, 50–59, and ≥ 60 years.

Sex: Male/female dichotomous scale.

Marital status: Single, married, divorced or widowed.

Employment status: Participants were grouped into two categories: employed or unemployed.

### Data analyses

Data collected was entered into an excel sheet and uploaded for analyses unto version 20 of the Statistical Package for Social Sciences (SPSS 20). Continuous data are presented as means ± SD as well as ordinate categories, and non-continuous data as proportions (%). Strengths of associations between categorical variables are presented as odds ratios and the differences between proportions determined with the aid of chi-squared tests (*X* ^2^). Differences between means of continuous variables when compared between groups were done with the aid of t-tests. Binary logistic regression permitted adjustments for potential confounders. All test statistics are two-sided and considered statistically significant at *p* < 0.05.

## Results

### Socio-demographic characteristics of the study population

A total of 104 participants were enrolled (50 hypertensive and 54 age- and sex- matched normotensive). The age of the participants ranged from 27 to 74 years with a mean age of 55.02 years (SD 8.69) and a sex ratio of 1.6 women for every 1 male (Table 1). No significant differences were noted between the cases and controls with regards to sociodemographic variables (Table 1).

**Table 1:** Socio-demographic characteristics of the study population

	Hypertension status				*p*-value	Entire study sample	
	Normotensives		Participants with hypertension				
	*N*	%	*n*	%		*N*	%
Variable	54	51.9	50	48.1		104	100%
Sex							
Male	21	38.9	19	38.0	0.926	40	38.5
Female	33	61.1	31	62.0		64	61.5
Age (years)							
Mean ± SD	55.24 ± 8.68		54.78 ± 8.79		0.789	55.02 ± 8.69	
25–49	10	18.5	10	20.0	0.974	20	19.2
50–59	28	51.9	26	52.0		54	51.9
≥ 60	16	29.6	14	28.0		30	28.8
Employment status							
Employed	27	50.0	20	40.0	0.306	47	45.2
Unemployed	27	50.0	30	60.0		57	54.8
Marital status							
Single	45	86.5	37	77.1	0.238	82	82.0
Married	7	13.5	9	18.8		16	16.0
Widow (ed)	0	0.0	2	4.2		2	2.0
Smoking							
Yes	0	0.0	0	0.0		0	0.0
No	54	100.0	50	0.0		104	100.0
Alcohol consumption							
Yes	4	7.4	3	6.0	0.775	7	6.7
No	50	92.6	47	94.0		97	93.3
On sleep medication							
No	54	100.0	50	100.0		104	100.0

### Anthropometric and physical examination findings of participants

Cases (patients with hypertension) had a significantly higher BMI, waist circumference as well as systolic and diastolic blood pressures, compared to their normotensive counterparts (Table 2).

**Table 2:** Anthropometric and physical examination findings of participants

Variable	Hypertension status				*p*-value	Entire study sample	
	Normotensives		Participants with hypertension				
	*N*	%	*N*	%		*n*	%
BMI (kg/m^2^)							
Mean ± SD	28.74 ± 4.25		31.35 ± 5.86		0.011	29.99 ± 5.23	
Normal BMI (18.5–25.00)	12	22.2	6	12.0	0.039	18	17.3
Overweight (25.01–29.99)	26	48.1	17	34.0		43	41.3
Obese (≥30.00)	16	29.6	27	54.0		43	41.3
Neck circumference (cm)							
Mean ± SD	35.92 ± 3.96		37.22 ± 4.20		0.106	36.54 ± 4.11	
Normal	14	23.1	10	20.0	0.474	24	23.1
Neck obesity	40	74.1	40	80.0		80	76.9
Waist circumference (cm)							
Mean ± SD	91.67 ± 10.11		98.32 ± 10.98		0.002	94.87 ± 11.00	
Normal	36	66.7	19	38.0	0.003	55	52.9
Abdominal obesity	18	33.3	31	62.0		49	47.1
Pulse (/min)							
Mean ± SD	71.46 ± 10.34		73.24 ± 14.43		0.464	72.32 ± 12.28	
Respiratory rate (cycles/min)							
Mean ± SD	17.65 ± 1.54		19.10 ± 7.25		0.154	18.35 ± 5.17	
Blood pressure (mm/hg)							
Systolic (Mean ± SD)	129.41 ± 7.68		153.08 ± 22.90		<0.001	140.79 ± 20.52	
Systolic BP raised?							
Yes	1	1.9	36	72.0	<0.001	37	35.6
No	53	98.1	14	28.0		67	64.4
Diastolic (Mean ± SD)						72.32 ± 12.28	
Diastolic BP raised?	77.76 ± 8.98		90.46 ± 12.40		<0.001		
Yes	3	5.6	34	68.0	<0.001	37	35.6
No	51	94.4	16	32.0		67	64.4
Clinical exam of other systems							
Normal	50	100.0	54	100.0		104	100.0

Conversely no significant differences were found between the two groups with regards to neck circumference, pulse rates and other physical examination findings (Table 2).

### Profile of hypertensive participants

Hypertensive cases were aged 54.78 ± 8.79 years on average and most had been diagnosed less than 5 years ago (mean duration since diagnosis of 4.46 ± 4.36 years); (Table 3). None of the hypertensive patients were on non-pharmacological (life-style changes) treatment only, compared to 20% who were on pharmacological treatment only and 80% on both. Almost all (96.0%) self-reported being compliant and just 24% had their blood pressure controlled at the time of the study (Table 3).

**Table 3:** Profile of hypertensive participants

Variable	*n*	%
	50	48.1
Number of years since diagnosis		
Mean ± SD	4.46 ± 4.36	
1–2	28	56.0
3–5	9	18.0
> 5	13	26.0
Current treatment strategy		
Non-pharmacological (lifestyle changes only)	0	0.0
Pharmacological only	10	20.0
Both	40	80.0
JNC classification of Hypertension		
Normal	00.	00
Pre-hypertension	15	30
Stage 1	17	34
Stage 2	18	36
Complaint to treatment (self-reported)		
No	2	4.0
Yes	48	96.0
Frequency of follow-up visits yearly		
Mean ± SD	3.94 ± 0.59	
1–2 visits	2	4.1
3 or more	47	95.9
Other medication on other than anti-HBP		
None	44	88.0
Statins	4	8.0
Warfarin	1	2.0
Other	1	2.0
Sleep medication use?		
No	50	100.0
HBP control		
Controlled BP (<140/90 mmHg)	12	24.0
Uncontrolled BP (>140/90 mmHg)	38	76.0

### Assessment of sleep patterns

High “risk”/likelihood of OSA was more common in hypertensive compared to normotensive participants (60% versus 20.4%, *p* < 0.001; OR 5.85, 95% CI 2.46–14.08); (Table 4). On adjusting for BMI the odds ratio of risk of poor sleep quality still remained high in participants with hypertension compared to normotensives (AOR 5.03; 95% CI, 1.90–13.33, *p* = 0.001).

**Table 4:** Sleep quality assessment in hypertensives compared to normotensives

	Hypertension status				*p*-value	Entire study sample		OR (95% CI)
	Normotensives		Participants with hypertension					
	*N*	%	*n*	%		*N*	%	
OSA assessment (BQ score)								
*Snoring*								
Yes	24	44.4	29	58.0	0.167	53	51.0	
No	30	55.6	21	42.0		51	49.0	
*No or Low “risk” of OSA*	43	79.6	20	40.0	<0.001	63	60.6	5.85
*High “risk” of OSA*	11	20.4	30	60.0		41	39.4	(2.46–14.08)
*Daytime sleepiness assessment (ESS score)*								
Mean ESS score	7.20 ± 3.32	7.58 ± 4.55	0.629	7.38 ± 2.36				
No sleep debt	42	77.8	42	84.0	0.421	84	80.8	
Sleep debt and daytime sleepiness	12	22.2	8	16.0		20	19.2	

No significant differences were found with regards to risk of OSA and resultant daytime sleepiness between hypertensive patients with controlled hypertension and those without as shown on the table below. On the other hand, resultant daytime sleepiness was non-significantly different between the two groups.

## Discussion

This study sought to conduct a preliminary assessment of the likelihood (risk) of obstructive sleep apnea (OSA) and resultant daytime sleepiness in hypertensive individuals compared to normotensive participants (through an age- and sex- matched case–control study design) in the cosmopolitan city of Yaoundé, Cameroon in order to contribute to fill knowledge gaps in sub-Saharan Africa on subtle consequences of hypertension such as sleep quality which affect patient quality of life and compliance to treatment.

Our findings reported a higher likelihood (“risk”) of OSA (60% versus 20.4%, *p* < 0.001; OR 5.85, 95% CI 2.46–14.08; Table 4) in hypertensive compared to normotensive persons with a five-fold odds ratio even after adjusting for BMI (AOR 5.03; 95% CI, 1.90–13.33, *p* = 0.001). These findings are similar to what was reported in another study [18]. The suggested mechanism to explain the link between OSA and hypertension (HTN) is fluid-shift which involves nocturnal rostral fluid shift from the legs [19] leading to reduced mean upper airway cross-sectional area. Intermittent hypoxia coupled to the reduced upper airway, triggers the chemo-reflex receptors which in turn stimulates the sympathetic nervous system leading to vasoconstriction and resultant increase in BP levels contrary to expected reduction in BP levels (termed nocturnal dipping). Patients with hypertension studies have shown, are more prone to increased rostral fluid shift results in airway narrowing (which may explain why OSA is more common in patients with hypertension) which further pushes up BP worsening the hypertension [19, 20]. Further lends support to the fact that that hypertension can contribute to worsening hypertension through the OSA pathway.

Another incriminated mechanism in the pathophysiological relationship between HTN and OSA involves Aldosterone excess [21–23], a hormone which assists in the regulation of circulating blood volumes and potassium concentration via a feedback loop within the adrenal cortex. In an observational study of consecutive participants with resistant HTN, participants were evaluated for primary hyperaldosteronism using plasma rennin activity <1.0 ng/mL/h and 24 h urinary aldosterone excretion >12 μg during high urinary sodium excretion (>200 mEq/24 h), and risk of sleep apnea with the aid of the Berlin Questionnaire [21]. Patients at high risk for OSA were almost two times more likely to have primary hyperaldosteronism (36% versus 19%, *p* < 0.05) with low plasma renin activity (1.2 ± 1.8 ng/mL/h versus 1.9 ± 4.1 ng/mL/h) and significantly greater 24 h urinary aldosterone excretion (13.6 ± 9.6 μg versus 9.8 ± 7.6 μg, *p* < 0.05) compared to participants at low risk of OSA, suggesting an association between primary hyperaldosteronism and OSA [21]. In another study by the same group, increased plasma aldosterone concentration and OSA were noted in patients with resistant HTN but not in control participants, suggesting that aldosterone excess may contribute to OSA severity [23]. These authors subsequently showed that OSA prevalence was significantly higher in participants with hyperaldosteronism compared with hypertensive patients with normal aldosterone levels (84% versus 77%). These findings suggest hyperaldosteronism may be a key factor in linking resistant HTN with OSA. These studies are, however, all observational and causality cannot be directly established.

Another proposed mechanism is the involvement of the structure and function of large arteries as well as ventricular remodeling. Hypertension is tightly linked to arterial stiffness (evaluated by pulse wave velocity) [12, 24–26]. In hypertensive patients, structural, mechanical or functional remodeling (geometric alterations in the vessel wall without a change in vessel volume) due to apoptosis, low-grade inflammation and vascular fibrosis reduce small arteries and arterioles lumen diameter (determinants of vessel elasticity). These lead to decreased vascular compliance (due vascular wall structural changes) and increased peripheral resistance and impaired endothelium-dependent vasodilation (mediated in part by nitric oxide, a potent vasoactive substance produced by vascular endothelium), common in hypertensive patients [26, 27]. Vascular endothelial dysfunction (arterial stiffness) increases intra-arterial pressure (evaluated by Pulse Wave Velocity) resulting in the transudation of plasma into the interstitial space which in turn favors overnight rostral fluid shift, OSA and intermittent hypoxia. These trigger the Sympathetic Nervous System to further increase blood pressure, heart rate and arterial constriction. OSA independently is known to be associated with increased arterial stiffness which explains why the coexistence of OSA and treated hypertension is associated with additive effects on decreased vascular compliance.

Evidence suggests that OSA is associated with left ventricular (LV) hypertrophy [12, 24, 26]. Heart remodeling associated with OSA can be explained by several mechanisms. Acutely, recurrent hypoxia may have a direct effect on the aorta, promoting reduction in the amount of glycosaminoglycans and collagen as well as at the level of the heart [25]. Obstructive apneas is also characterized by negative intrathoracic pressure against an occluded upper airway, leading to LV transmural pressure increase (an important determinant of LV afterload). Cyclic increases in the BP due to apnea also contribute to increase LV afterload. These limit the aorta and proximal large vessels from serving as an elastic buffering chamber (the Windkessel function) which can store as much as 50% of the LV stroke volume during systole, leading to increased LV mass and LAD [25].

Hypertension on its part is not only associated with increased arterial stiffness but also with early signs of heart remodeling manifesting as increased Left Atrial Diameter (LAD), Interventricular Septal Wall Thickness (IVST), Left Ventricular Posterior Wall Thickness (LVPWT), LV mass index and percentage of LV hypertrophy as well as heart remodeling.

Anthropometric parameters such as BMI and neck circumference have equally been shown to affect sleep patterns in people including hypertensive patients. In our study, hypertensive participants were significantly more obese than normotensive participants. Despite adjusting for BMI with the aid of multivariate logistic regression analyses, hypertensive participants were 5 times more likely to have having a greater “risk” of OSA than normotensive participants [AOR: 5.03; 95% CI: 1.90–13.33, *p* = 0.001]. This was similar to what was reported in another study that studied the association between hypertension and risk of OSA assessed with the aid of the Berlin Questionnaire [28].

Though the prevalence of snoring (assessed with the aid of the Berlin Questionnaire was higher in participants with hypertension compared to normotensives (58.0% versus 44.0% respectively), the difference was not significant, *p* = 0.167); Table 4. This was inconsistent with the results of a similar study which revealed a significantly higher prevalence of snoring in participants with resistant hypertension [29]. This discrepancy could be explained by our relatively small sample size compared to the latter study.

This study showed controlled hypertensive participants (hypertensive patients on anti-hypertensive treatment with BP <140/90 mmHg) had a relatively lower risk of OSA (50% versus 63.2%) than those with uncontrolled hypertension (Table 5), which difference was however not significant (*p* = 0.718). Our relatively small sample size may explain the absence of a significant difference for adequate BP control may lead to a relatively better sleep quality via a reduction in nocturnal rostral fluid shift (proportional to the amount of fluid displaced from the legs) which as explained above has been postulated in participants with hypertension with resultant reduction of mean upper airway cross-sectional area. This is supported by literature which suggests upper airway reduction literature is significantly more pronounced in the patients with uncontrolled hypertensive compared with controlled hypertensive participants [20, 30]. Further studies are required to further investigate this relationship.

**Table 5:** Sleep parameters with respect to level of BP Control

Variable	Hypertension Control				*p*-value	Entire Hypertensive Sample	
	Controlled Hypertension		Uncontrolled Hypertension				
	*N*	%	*N*	%		*N*	%
	12	24.0	38	76.0		50	100.0
OSA assessment (BQ score)							
*Snoring*							
Yes	6	50.0	23	60.5	0.520	29	58.0
No	6	50.0	15	39.5		21	42.0
*Low “risk” of OSA*	6	50.0	14	36.8	0.417	20	40.0
*High “risk” of OSA*	6	50.0	24	63.2		30	60.0
*Daytime sleepiness assessment (ESS score)*							
Mean ESS score	8.00 ± 4.79	7.45 ± 4.54	0.718	7.58 ± 4.55			
No sleep debt	10	83.3	32	84.2	1.00	42	84.0
Sleep debt and daytime sleepiness	2	16.7	6	15.8		8	16.0

We also did not observe a trend increasing risk of OSA with respect to the BP severity (assessed as per JNC classification of hypertension severity). This absence of a trend may be due to our study sample size. Larger scale studies are needed to further investigate the relationship between OSA and hypertension severity.

Resultant daytime sleepiness (measured by Epworth Sleepiness Score) was not significantly different between cases and normotensive participants. This finding is similar to that reported by other studies [27, 29–31]. The absence of a difference may be as a result of repeated sympathetic nervous system firing during the day which maintains an alert and awake state.

All the hypertensive participants in our sample were on anti-hypertensive drugs (a mean of 2 drugs per person). Amongst these participants, 12 (24%) had well controlled BP (<140/90 mmHg) as recommended by the European Society of Hypertension (ESH) and the Joint National Committee on prevention, detection, evaluation and treatment of high blood pressure [9, 10] as opposed to 38 (76%) who were not controlled (>140/90 mmHg). This proportion of controlled hypertension though higher than that of previous blood pressure control studies in Cameroon [32, 33], is lower than that obtained by Katte and collaborators in a similar setting and on a larger sample [34] as well as rates reported in the US as part of the National Health and Nutrition Examination Survey (NHANES) [8].

The tendency for persons with hypertension to seek treatment is the result of the efforts of several screening and health education campaigns. Significantly greater mean BMI, mean neck circumference and mean waist circumference among the uncontrolled HBP cases compared to those with controlled BP could possibly link account for the poor BP control.

This study has some limitations that should be considered in designing future sleep studies. First, its cross-sectional study design gives only a snapshot and its results may be different or more conclusive if a prospective design had been chosen. The relatively small sample size might explain the unintentional absence of smokers in the study sample, which affects the study’s generalizability to the general Cameroonian population where 1 in every 9 persons smokes [34]. This weakness is however a strength and suggests that the observed high likelihood of obstructive sleep apnea in patients with hypertension compared to controls, is likely due to the pathological changes explained above, observed in hypertension and not (directly) smoking, a known risk factor for sleep apnea. Sleep questionnaires though user-friendly, easy to use and cheaper, can be subjective tools in assessing sleep compared to other methods like polysomnography and actigraphy. This was minimized by coaching participants on filling the sleep questionnaires. Studies are needed to determine the sensitivity, specificity and validity of these sleep questionnaires in our settings, as quick and reliable tools to detect disordered sleep, by comparing them with gold standards. Finally this pilot study involved a small hospital-based sample. The weakness of hospital data can best be dealt with by conducting large and community-based studies which time and resources could not permit. However, the study’s hospital-based participants were age-and-sex matched with community-based controls to improve quality.

## Conclusions

This study provides preliminary data on sleep disorders in a sample of hypertensive participants in a sub-Saharan situation. The higher prevalence of the risk of OSA in hypertensive compared to normotensive participants is worth noting. Further larger scale and prospective studies however need to be carried out using detailed investigations (actigraphy, polysomnography) and biological markers to shed more light on the pattern of sleep in participants with hypertension in our setting as opposed normotensives and how it impacts blood pressure values. More emphasis on subtle consequences of hypertension like sleep perturbation should be given during follow-up of patients with hypertension so as to limit its impact on treatment adherence, disease progression and quality of life. Sleep questionnaires validated for use in our setting against gold standards could be used in the clinical settings as simple and quick tools to determine those at risk and in need for further investigation.

## Abbreviations

BP: Blood pressure
BQ: Berlin Questionnaire
DBP: Diastolic blood pressure
ESS: Epworth Sleepiness Scale
HBP: High blood pressure
HTN: Hypertension
JNC: Joint National Committee
LV: Left ventricle
OSA: Obstructive sleep apnea
SBP: Systolic blood pressure
SDB: Sleep Disordered Breathing
